# GC-MS analysis of an ethanolic extract of *Ocimum* species: a network pharmacology analysis insight towards obesity

**DOI:** 10.2144/fsoa-2023-0202

**Published:** 2024-05-15

**Authors:** Priyadharshini Gnanamurthy, Manoj K Narasimhan, Sarvesh Sabarathinam

**Affiliations:** 1 Department of Genetic Engineering, School of Bioengineering, College of Engineering & Technology, SRM Institute of Science & Technology, Kattankulathur, Chennai, Tamil Nadu, 603203, India; 2 Drug Testing Laboratory, Interdisciplinary Institute of Indian System of Medicine (IIISM), SRM Institute of Science & Technology, SRM IST, Kattankulathur, Chennai, Tamil Nadu, 603203, India

**Keywords:** GC-MS, molecular docking, network pharmacology, obesity, *Ocimum* species

## Abstract

**Aim:** In this study, we have selected two different *Ocimum tenuiflorum* plants, *Ocimum tenuiflorum* (Rama tulsi) (OTRT) and *Ocimum tenuiflorum* (Krishna tulsi) (OTKT). **Materials & methods:** In the present investigation, ethanol was used as a solvent to estimate the bioactive compounds present in it through gas chromatography-mass spectrometry (GC–MS). **Results:** Based on the GC-MS data benzenepropanoic acid, 3-methoxy-alpha,4-bis[(trimethylsilyl)oxy was found to be the potent compound in OTRT (MW: 428.74 g/mol) and methyl 3-(4-benzyloxy-3,5-dimethoxyphenyl)-2-methylpropanoate in OTKT (MW: 342.39 g/mol). To estimate its pharmacological application, an integrated Network Pharmacology approach is performed toward the disease target obesity. **Conclusion:** From the protein–protein interaction from the string database, SRC, BCL2, EGFR, MTOR, CDK1, ERBB2, MAPK1, FYN, AR and MAPK14 are the top-ranked targets.

## Abstract (plain language summary)

*Ocimum tenuiflorum* species, such as Tulsi or Holy Basil, are traditionally known for their potential medicinal properties.

They are reported to contain various bioactive compounds, including steroids, tannins, alkaloids, flavonoids and phenolics, and have demonstrated anti-hyperglycemic properties, making them valuable for diabetes management.

Network biology and network pharmacology were employed to explore potential targets for obesity management.

Samples of two *Ocimum* tenuiflorum species, Rama tulsi and Krishna tulsi, were collected, authenticated, and prepared for analysis.

The samples were subjected to GC-MS analysis to identify compounds of interest.

Two high molecular weight compounds, OTRT and OTKT, were selected for further study.

Network pharmacology was used to identify potential targets related to obesity for these compounds.

GC-MS analysis revealed various compounds, with OTRT and OTKT being selected due to their high molecular weight.

Network pharmacology identified key targets associated with obesity and potential interactions with the selected compounds.

The study highlighted the importance of kinase enzymes in various cellular processes related to obesity.

The KEGG pathway analysis provided insights into the potential mechanisms of action of the compounds.

The drug-likeness and pharmacokinetic profiles of OTRT and OTKT were assessed.

Molecular docking analysis indicated the binding affinity of these compounds to specific targets.

The study emphasized the potential toxicity and pharmacological properties of the compounds.

The study successfully identified bioactive compounds from *Ocimum tenuiflorum* species through GC-MS analysis.

Network pharmacology provided valuable insights into potential targets for obesity management.

The findings suggest that these compounds could serve as lead molecules in drug development for obesity and diabetes management.

Further preclinical and clinical investigations are needed to validate the potential therapeutic applications of these bioactive compounds.

## Graphical abstract

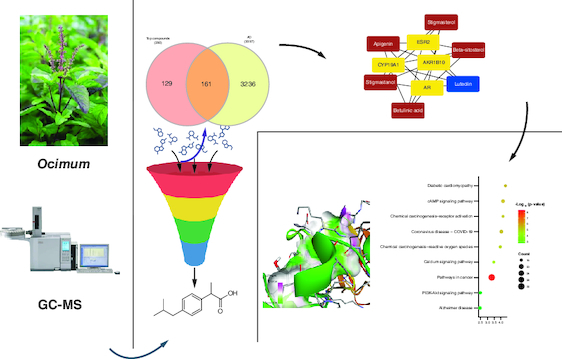

*Ocimum tenuiflorum* species are herbal plants that are majorly available in the Asian region, which belongs to the Lamiaceae family. More than 100 species of *Ocimum tenuiflorum* have been reported so far, and the *Ocimum tenuiflorum* species have reported multiple pharmacological effects. Due to the presence of steroids, tannins, alkaloids, flavonoids and phenolics [1]. Many regions, including Asia and Africa, plant *O. tenuiflorum* L., known as Tulsi or Holy basil. By boosting the insulin secretion from isolated islets, perfused pancreas and clonal pancreatic cells, the leaf extracts of *O. tenuiflorum* L. (formerly known as *O. sanctum* L.) have been demonstrated to exhibit anti-hyperglycemic properties. It is regarded as one of the most effective traditional plants for treating diabetes and comes highly recommended [2]. Some studies suggest that extracts from certain *Ocimum* species, such as holy basil, may have the ability to lower blood sugar levels. They are believed to influence insulin secretion and sensitivity, potentially helping people with diabetes maintain better control over their blood glucose levels. *O. tenuiflorum* extracts reported to have anti-diabetic activity due to its ability to suppress endogenous glucose release, inhibit glycogenolysis and/or stimulate glycogenesis [3,4]. Many pre-clinical and *in silico* studies shows the possible therapeutic potential of *O. tenuiflorum* in the management of diabetes [5]. Network biology and Network Pharmacology is an integrated approach which helps in the identification of druggable genomes [6]. Various genes play a significant role in obesity and other cardiometabolic complications, either directly or indirectly, such as SRC. The SRC gene is involved in regulating adipocyte differentiation and energy expenditure, influencing obesity risk. MTOR is a central regulator of metabolism and adipogenesis, contributing to the development of obesity when dysregulated. EGFR signaling can affect appetite regulation and adipose tissue expansion, playing a role in obesity. The *BCL2* gene influences apoptosis and adipocyte survival, impacting obesity-related adipose tissue changes. CDK1 contributes to cell cycle control and adipogenesis, affecting adipocyte development in obesity. ERBB2 signaling can influence adipocyte growth and energy balance, potentially contributing to obesity [^7-11^]. MAPK1 participates in MAPK signaling pathways associated with inflammation and obesity-related complications. The FYN kinase is implicated in central leptin signaling, affecting appetite control and obesity development. The androgen receptor (*AR*) gene can impact fat distribution and body composition, influencing obesity risk. *MAPK14* is involved in inflammatory pathways and can contribute to obesity-related inflammation and insulin resistance [11,12]. Therefore, in this paper, we aid to discuss the GC-Ms analysis of two different *Ocimum tenuiflorum* plants, *Ocimum tenuiflorum* (Rama tulsi) (OTRT) and *Ocimum tenuiflorum* (Krishna tulsi) (OTKT) and integrated network pharmacology approach toward obesity in the identification of top-ranked targets.

## Method

### Sample collection

The whole plants of two different varieties of *Ocimum tenuiflorum* species, *Ocimum tenuiflorum* (Rama tulsi) and *Ocimum tenuiflorum* (Krishna tulsi), were collected from Villupuram, Villupuram district, Tamil Nadu, India during the summer season. M. Sivaraman, PG and Research Department of Botany, Arignar Anna Government Arts College, Villupuram, Tamilnadu, India, verified the authenticity of the plants. The leaf, stem and root part of the two species were separated carefully, washed in running water and then kept for air drying at room temperature. The air-dried samples were powdered using an electric blender and stored in airtight containers with proper labeling.

### Preparation of sample

Five g of powdered material from the whole plants of two *Ocimum tenuiflorum* species were carefully weighed using a precision scale. They subsequently underwent an extraction process involving 50 ml of ethanol within a 100 ml conical flask. The entire plant extracts were placed in a shaking orbital incubator for a duration of 72 h to ensure thorough extraction. To guarantee complete extraction, a re-extraction step was performed for each sample using ethanol until a colorless solvent was obtained. Following the completion of the extraction process, the resulting fraction was filtered using a Whatman no. 1 size filter and concentrated through the use of a rotary evaporator. The concentrated extracts were then air-dried in glass petriplates to facilitate the removal of residual ethanol via evaporation. In total, six air-dried extracted samples from the two *Ocimum tenuiflorum* species were collected, weighed and subsequently utilized for GC-MS analysis.

### GC-MS analysis

**Figure 1.**
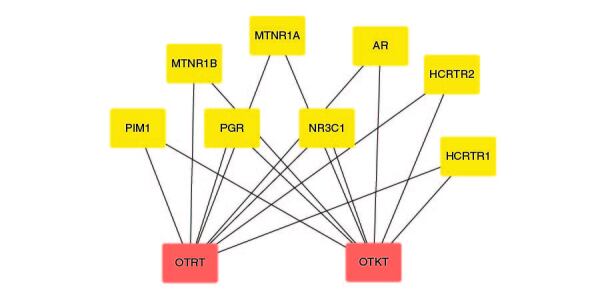
The top-ranked targets related to *Ocimum tenuiflorum* (Rama tulsi) and *Ocimum tenuiflorum* (Krishna tulsi).

The extracted samples from the whole part of the two *Ocimum tenuiflorum* species (Rama and Krishna tulsi) were subjected to GC-MS analysis using GC-MS QP 2010 by Shimadzu. The sample preparation for GC-MS analysis was enacted by the addition of 1 mg of the extract sample with 1 ml of ethanol as a solvent. The instruments was equipped with a split mode injector, through which the sample was injected into the Zebron ZB-FFAP column fused with silica, with the dimensions of 60 m × 0.25 mm and a film thickness of 0.25 μm for separations. Helium was used as a carrier gas, flowing at a rate of 1 ml/min. The sample was injected in a split mode at the rate of 1 μl/min with a split ratio of 1:50. The injection temperature was kept at 250 °C. The other conditions in operation of GC-MS includes ion source temperature, 200 °C; interface temperature, 280 °C; pressure, 53.5 kPa. The column oven temperature was kept at 50°C and maintained for 1 min, it was then increased gradually at 10 °C/min until it reached 280 °C, and was then held at this temperature for 2 min. The GC-MS scan spectra obtained at the range width of 40–700 m/z. Wiley 8 and NIST 14 libraries attached with the GC-MS instrument were used to identify the components by comparing their retention indices. The identified components based on the computer libraries (Wiley 8 and NIST 14) were tabulated. The GC-MS data of OTRT is given in Table 1 and the GC-MS data of OTKT is given in Table 2. For the Network analysis the targets related to OTRT and OTKT were estimated from Swiss target Online database. The top-ranked targets related to OTRT and OTKT were given in Figure 1.

**Table 1.** GC-MS analysis of *Ocimum tenuiflorum* (Rama tulsi).

Compound	Molecular weight	Molecular formula	RL	
			Peak %	RT
2-methoxy-4-vinyl phenol	150.17 g/mol	C9H10O2	1.12	11.846
Phenol, 2-methoxy-4-(2-propenyl)-	206.23 g/mol	C12H14O	16.73	12.440
Guanosine	283.24 g/mol	C10H13N5O5	10.03	13.619
Beta.-d-glucopyranose,1,6-anhydro-	162.14 g/mol	C6H10O5	2.60	14.300
Neophytadiene	278.51 g/mol	C20H38	5.92	18.306
9,12,15-octadecatrienoic acid,(z,z,z)-	278.42 g/mol	C18H30O2	3.38	21.447
Hexadecanoic acid	256.42 g/mol	C_16_H_32_O_2_	1.10	21.594
Phytol, acetal	338.56 g/mol	C22H42O	1.45	22.127
Octanoic acid, 2-dimethylaminoethyl ester	215.33 g/mol	C10H20O2	1.23	22.764
3-cyclopentylpropionic acid, 2-dimethylaminoethyl ester	213.32 g/mol	C14H27NO2	3.08	24.363
Tofisopam	382.5 g/mol	C20H26N2O	1.25	25.305
Butanoic acid, ethyl ester	116.15 g/mol	C6H12O2		
Propane, 1-(1-ethoxyethoxy)-	132.20 g/mol	C7H16O2		
1-butanol, 3-methyl-, acetate	130.18 g/mol	C7H14O		
6-(iodomethyl)-6-methyl-4-oxahexanolide	162.19 g/mol	C20H30O		
1,2,5-thiadiazol-3-amine, 4-phenyl-	177.23 g/mol	C8H7N3S		
n-(2,6-dimethyl-phenyl)-n-(2-morpholin-4-yl-2-phenyl-acetyl)-benza	197.66 g/mol	C20H18O4		
2-(1′-hydroxy-3′-phenyl-(e)-prop-2′-enyl)-1,3-dibenzyl-4,5-dimethyl-1,	231.54 g/mol	C11H14O2		
9(10h)-anthracenone, 10-ethoxy-, oxime	145.97 g/mol	C20H14O2		
4′-(2-acetoxyethoxy) acetophenone	222.24 g/mol	C12H14O4		
Benzenepropanoic acid, 3-methoxy-alpha,4-bis[(trimethylsilyl)oxy	428.74 g/mol	C19H36O5SI3		

**Table 2.** GC-MS analysis of *Ocimum tenuiflorum* (Krishna tulsi).

Compound	Molecular weight	Molecular formula	KL	
			Peak %	RT
4h-pyran-4-one, 2,3-dihydro-3,5-dihydroxy-6-methyl-	144.25 g/mol	C6H8O4	1.00	9.182
Guanosine	283.24 g/mol	C5H54N5O	8.40	13.660
(e)-labda-8(17),12-diene-15,16-dial	302.45 g/mol	C20H30O	1.11	17.331
4-((1e)-3-hydroxy-1-propenyl)-2-methoxyphenol	180.20 g/mol	C10H120	1.04	17.420
Neophytadiene	278.52 g/mol	C20H38	4.33	18.308
N-hexadecanoic acid	256.42 g/mol	C16H32O	4.21	19.608
Phytol	296.53 g/mol	C20H40O	4.86	21.187
9,12,15-octadecatrienoic acid, (z, z, 3z)-	278.42 g/mol	C18H30O	5.39	21.453
Phytol, acetate	338.57 g/mol	C22H42O2	2.17	22.130
3-cyclopentylpropionic acid, 2-dimethylaminoethyl ester	213.32 g/mol	C12H23NO2	1.11	24.363
Propionic acid, 2-mercapto-, allyl ester	146.21 g/mol	C6H10O2S		
Oxirane, 2,3-dimethyl-, *cis*-	72.10 g/mol	C4H8O		
Propane, 1,1-diethoxy-	132.20 g/mol	C7H16O		
Propane, 1-(1-ethoxyethoxy)-	132.20 g/mol	C7H16O2		
2-[(2-propenyl) oxy] ethanol	102.13 g/mol	C5H10O2		
2,2-dibenzyl-1,3-thioxane	256.34 g/mol	C17H20O2		
Methyl 3-(4-benzyloxy-3,5-dimethoxyphenyl)-2-methylpropanoate	342.39 g/mol	C20H22O5		
Mepronil	269.34 g/mol	C17H19NO2		
7-methoxy-3-(p-methoxyphenyl)-4h-chromen-4-one	284.31 g/mol	C18H16O4		
Naphtho[2,1-b]furan-6-methanol, dodecahydro-6,9a-dimethyl	236.39 g/mol	C16H28O		
Cyclopropylmethanol	72.11 g/mol	C4H8O		
2,5-dihydro-1h-pyrrole	69.10 g/mol	C4H7N		

## Results

**Figure 2.**
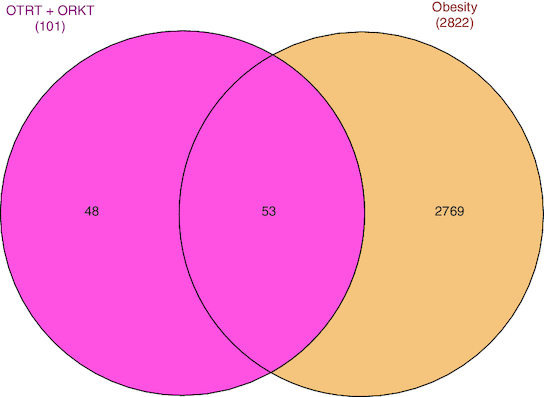
Overlapped targets of *Ocimum tenuiflorum* (Rama tulsi) and *Ocimum tenuiflorum* (Krishna tulsi) and obesity-related target.

**Figure 3.**
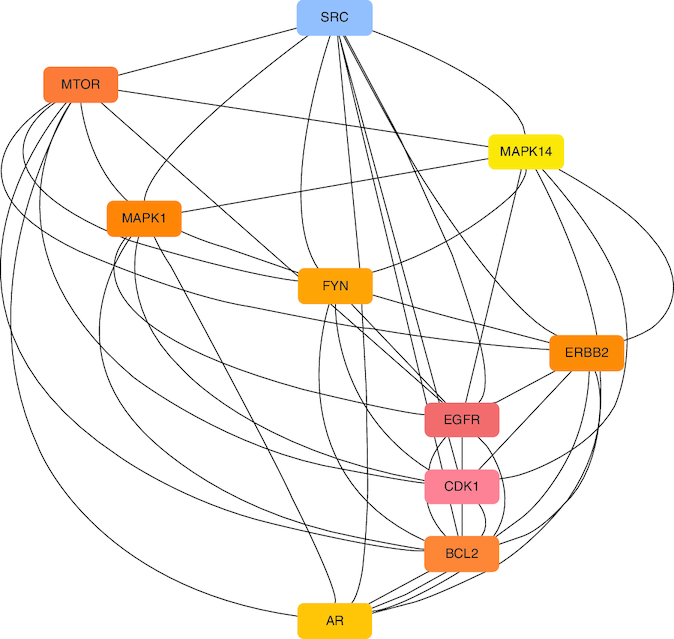
TOP-ranked targets of PPI network.

**Figure 4.**
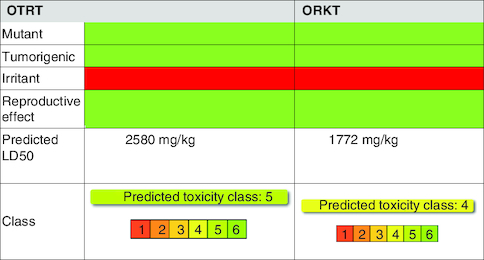
Toxicity profile of *Ocimum tenuiflorum* (Rama tulsi) and *Ocimum tenuiflorum* (Krishna tulsi).

The GC-MS analysis reported the presence of many compounds. Of those, only two compounds reported with a high molecular weight. The identified compounds are provided in Table 1. The selected compound from Rama tulsi was coded as OTRT–Benzenepropanoic acid, 3-methoxy-alpha,4-bis[(trimethylsilyl)oxy(C19H36O5SI3) (MW: 428.74 g/mol). The selected compound from Krishna tulsi was OTKT: Methyl 3-(4-benzyloxy-3,5-dimethoxyphenyl)-2-methylpropanoate (C20H22O5) (MW: 342.39 g/mol). The GC-MS data is given Tables 1 & 2. Based on the higher molecular weight justification these two compounds further taken in to network pharmacology. From the GC-MS data, major compounds have been securitized for network pharmacology. In this study, 01 compounds have selected for *Ocimum tenuiflorum* (Rama tulsi) (OTRT) based on the GC-MS data given in table 1 and 01 compounds were selected from *Ocimum tenuiflorum* (Krishna tulsi) (OTKT) based on the GC-MS data given in table 2. The list of selected compounds was given in Table 3. The drug-likeness profile and pharmacokinetic profile of OTRT and OTKT is given in Table 4. The top-ranked targets of selected compounds were illustrated in Figure 1. The toxicity profile is given in Figure 4. The obesity (Obesity, C0028754) disease-related targets were generated from the Disgenet database. The obesity-related targets and OTRT and OTKT were overlapped and overlapped genes were identified as hub-genes for protein–protein interaction (PPI). The overall target numbers are illustrated in Figure 2. The PPI interaction was estimated from string online database the top-ranked targets were illustrated in hierarchy model in Figure 3. SRC, BCL2, EGFR, MTOR, CDK1, ERBB2, MAPK1, FYN, AR and MAPK14 are the top-ranked targets (high probable drug active sites) detected in the network construction. Kinase enzymes play a crucial role in various cellular processes, including signal transduction and metabolic regulation. In the context of obesity, several kinases have been studied for their involvement in the development and progression of obesity. The molecular docking report of OTRT and OTKT toward PDB:5JSN is given in Table 5.

**Table 3.** List of compounds selected based on GC-MS analysis.

Compound name	Molecular weight	Molecular formula
Benzenepropanoic acid, 3-methoxy-alpha,4-bis[(trimethylsilyl)oxy 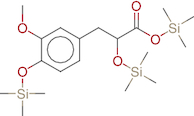	428.74 g/mol	C19H36O5SI3
Methyl 3-(4-benzyloxy-3,5-dimethoxyphenyl)-2-methylpropanoate 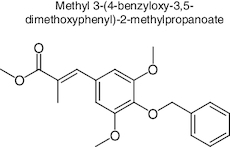	342.39 g/mol	C20H22O5

**Table 4.** Drug-likeness profile and pharmacokinetic profile of *Ocimum tenuiflorum* (Rama tulsi) and *Ocimum tenuiflorum* (Krishna tulsi).

OTRT	OTRT	OTKT
Lipinski	Yes; 0 violation	Yes; 0 violation
Ghose	Yes	Yes
Veber	Yes	Yes
Egan	Yes	Yes
Muegge	No; 1 violation: XLOGP3>5	Yes
Bioavailability score	0.55	0.55
GI absorption	High	High
BBB permeant	Yes	Yes
P-gp substrate	Yes	No
CYP1A2 inhibitor	No	Yes
CYP2C19 inhibitor	No	Yes
CYP2C9 inhibitor	No	Yes
CYP2D6 inhibitor	Yes	Yes
CYP3A4 inhibitor	No	Yes

**Table 5.** Docking report of *Ocimum tenuiflorum* (Rama tulsi) and *Ocimum tenuiflorum* (Krishna tulsi) toward PDB:5JSN.

OTRT with PDB:5JSN	ORKT with PDB:5JSN
-5.2 kcal/mol	-5.6 kcal/mol
LYS22 ARG26 ASP102 SER105 ARG106 ARG109 PHE112 ALA113 SER116 GLU152 VAL156 VAL159 GLU160 ASN163	YS5 LEU8 ASP9 LYS12 ARG92 LEU96 ASP3 PRO4 LYS5 GLN85 GLU88 ARG92
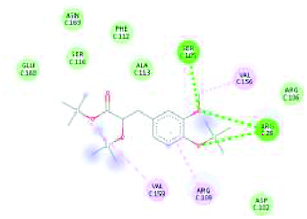	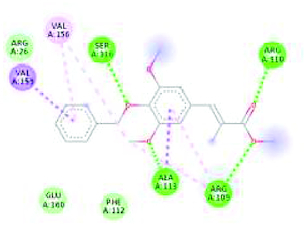
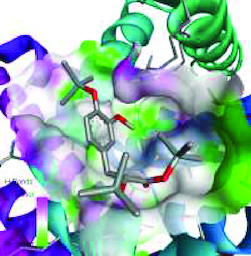	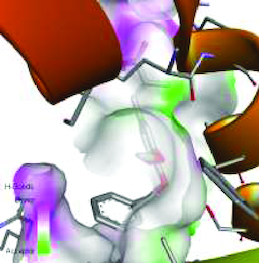

**Figure 5.**
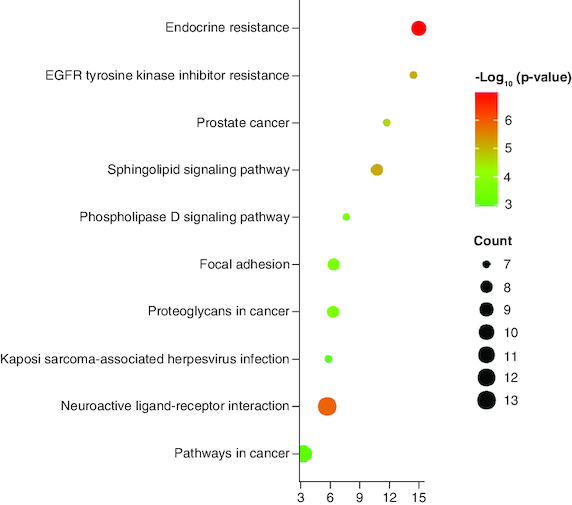
KEGG pathways of the hub-genes.

The KEGG pathways was estimated from the hub-genes. The KEGG pathway bubble chart is depicted in Figure 5. The drug-likeness profile and pharmacokinetic profile of the selected compounds were given Table 2. The molecular docking analysis was performed for the BCL2 target PDB: 5JSN for both of the compounds. The docking score of OTRT is -5.2 kcal/mol and OTKT is -5.6 kcal/mol. The molecular docking analysis shows the impact of hydrogen bond acceptor on the certain amino residues.

## Discussion

The Network Pharmacology approach helps in the estimation of multiple targets [13,14]. The computer aided drug discovery is estimating the drug pharmacokinetic parameters. In this study, OTRT and OTKT is high in absorption and having the strong tendency of penetrating the blood–brain barrier. OTRT is CYD26 inhibitor and OTKT is inhibitor of CYP1A2, CYP2C19, CYP2C9, CYP2D6 and CYP3A4. The OTRT falls under class 5 toxicity and OTKT is class 4. Both of the compounds seems highly toxic for irritant effect [15,16]. Another study showed that aqueous extract of *O. sanctum L.* exhibited a significant decrease in the blood glucose level (p < 0.000 1) [2]. Ezeani C *et al.* observed that the treatment with 100 and 200 mg/kg extract significantly reduced fasting blood glucose concentration and slightly increased mean body weight in treated groups [17]. Khalil H E *et al.* observed that total methanolic extract of *Ocimum tenuiflorum* speices distinctly diminished the mRNA and protein expression of NF-κB, cleaved caspase-3, and BAX, and increased BCL2 expression (reflecting its protective and antiapoptotic actions) [18]. Developing effective anti-diabetic drugs with no side effects is required in this contemporary state.

## Conclusion

The present investigation focused on identifying various bioactive compounds from the leaves of two *Ocimum tenuiflorum* species by GC–MS analysis. The network Pharmacology report shows the multiple-active sites of major bioactive compounds. This integrated network pharmacology approach may help identify lead molecules in drug discovery at the early stages of drug development. Further preclinical and clinical investigation is warranted to support the selected bioactive compounds in clinical practice.
